# Risk factors in Hymenoptera venom allergy 

**DOI:** 10.5414/ALX01320E

**Published:** 2017-08-04

**Authors:** F. Ruëff, J. Kroth, B. Przybilla

**Affiliations:** AllergieZentrum, Klinik und Poliklinik für Dermatologie und Allergologie, Ludwig-Maximilians-Universität, Munich, Germany

**Keywords:** specific immunotherapy, mastocytosis, tryptase, honey bee venom, wasp venom, risk factor

## Abstract

Abstract. Risk factors should be part of the decision, of which patient should be offered venom immunotherapy (VIT) and how VIT should be performed. Risk factors for a severe systemic anaphylactic reaction (SAR) after a Hymenoptera field sting include a preceding less severe sting reaction, a wasp sting, an increased baseline serum tryptase concentration (BSTC), mastocytosis, older age, ACE inhibitor medication, and male gender. During VIT, treatment with honey bee venom is the most important risk factor for a SAR. Further risk factors include a high BSTC (for vespid VIT only), presence of venom specific IgE in serum, any antihypertensive medication during therapy, and an ultra-rush protocol for build-up. Treatment failure is more common in patients suffering from honey bee venom allergy, high BSTC (for vespid VIT only) or mastocytosis, and in those who had experienced side effects during VIT. Besides discontinuing antihypertensive medication or switching to a moderate type of dose increase during build-up, little can be done to minimize the risks associated with VIT. Increasing the maintenance dose may improve the efficacy of VIT. In patients with a particularly high risk for treatment failure, or in case of treatment failure, VIT should include an increased maintenance dose right from the beginning. Usually, 200 µg will be sufficient.

German version published in Allergologie, Vol. 33, No. 7/2010, pp. 297-302

## Introduction 

It is essential to know the risk factors in patients with systemic anaphylactic reactions (SAR) to Hymenoptera venom in order to be able to decide for which patients venom immunotherapy (VIT) is indicated. The knowledge about risk factors can also influence the decision on individual treatment schemes or precautions. Concerning the duration of a VIT, the latest EAACI (European Academy of Allergy and Clinical Immunology) guideline has also laid more stress on individual risk factors so that the decision for VIT discontinuation should be made on an individual basis [1]. 

## Risk for systemic sting reactions 

The sensitization to Hymenoptera venom is a necessary but not sufficient precondition for the occurrence of an IgE-mediated SAR after a Hymenoptera sting. Usually sensitization against Hymenoptera venom is caused by Hymenoptera stings. Table 1 shows a list of activities that carry a particularly high risk for Hymenoptera stings. Sensitization against Hymenoptera venom is relatively frequent in the general population: in approximately 25% of adults and up to 50% of children specific IgE antibodies in the serum or positive immediate-type skin reactions to Hymenoptera venom can be detected [8, 32, 34], and approximately 60% of beekeepers show honey bee venom-specific IgE antibodies [20]. If the sensitization to Hymenoptera venom is caused by cross-reactivity against structurally similar allergens from other allergen sources, certain cross-reactive carbohydrate determinants (CCD) can be considered as a potential source. In plants CCD are common pan epitopes, but they are also found in Hymenoptera venom [10, 11, 22]. 

In most cases a sensitization to Hymenoptera venom is clinically not relevant, as only approximately 3% of the general population ever experience an IgE-mediated SAR after a honey bee or wasp sting [34]. The low clinical relevance of the sensitization to Hymenoptera venom will probably be due to the fact that most people live in urban areas where they are only rarely exposed to Hymenoptera stings. In beekeepers SAR have been reported in 14 – 32% of cases [20], suggesting that a high exposure to stings represents a risk factor for SAR. Nevertheless, a contrary effect was described in beekeepers: for beekeepers who were stung extremely frequently (> 200 stings/year) no SAR were reported [2]. In this context it could be discussed if this reflects a selection effect or if the high allergen dose induces a natural tolerance. 

There are no prospective follow-up studies for the general population examining the risk for SAR following Hymenoptera stings. Retrospective studies analyzing cases of SAR following a Hymenoptera sting showed that the following factors could increase the risk:

- patient history with increased local sting reaction [24],
- short time period after an earlier (tolerated) sting [24].

Such retrospective studies might, however, have a recall bias, that means that patients attach more importance to earlier events when a disease occurs, which leads to a bias in the patient history. 

Sting provocations with honey bees in persons with increased exposure to bee stings but without SAR to stings in their history showed that a higher concentration of Hymenoptera venom-specific IgE antibodies in the serum [6] is a predictive factor for SAR. 

## Severity of sting reactions 

Allergic reactions to Hymenoptera stings can be more or less severe. This is not only true for the comparison between different patients, the stage of severity can also vary in each individual patient (Table 2). All data on the analysis of risk factors for sting reactions and the corresponding stages are based on retrospective studies. Particularly for skin test reactions and for venom-specific serum IgE antibodies it has to be taken into account that the findings have been obtained after the sting event and that therefore no conclusions about their influence in a previous event can be drawn. The following risk factors for a severe sting reaction have been described:

- Wasp stings are associated with more severe reactions than honey bee stings [14, 29, 35].
- Increased BSTC [12, 16, 29, 33]: the risk-increasing effect of an elevated BSTC develops already within the normal range (95th percentile < 11.4 µg/l) [29].
- Cutaneous and/or systemic mastocytosis [23, 28].
- Older age [29].
- Angiotensin-converting enzyme (ACE) inhibitors [29].
- In general, preceding systemic sting reactions seem to have a booster effect and to predispose the patient to later severe sting reactions [14, 29, 35]. Nevertheless some patients tolerate later stings despite severe reactions to preceding stings. The risk for untreated patients to develop a SAR again when further stings occur is between 10 and 60%, depending on the patients studied [26].
- Male gender [14, 29]. In this context a selection bias could exist, as many men tend to visit a physician only in severe cases or advanced diseases. On the other hand, as some of the activities that carry a higher risk to be stung are more frequently performed by men, gender could be a real risk factor due to the higher exposure.

Although b-blockers have not been proven to be a risk factor for severe anaphylaxis of different origin [5] or in Hymenoptera venom allergy [29], they should – as far as possible – be discontinued; if therapy with b-blockers is inevitable, a cardioselective agent should be used. ACE inhibitors, on the other hand, should be discontinued. 

According to the current EAACI recommendations [1] in patients with sting reactions Grade I an indication for VIT only exists, when further risk factors are present or when the patient’s quality of life is impaired due to the Hymenoptera venom allergy. As frequent sting events carry the risk of an increase in severity, in adults VIT should be carried out independently of the severity. Only for children aged 2 – 16 years it could be shown that in cases of SAR that were limited to the skin further sting events lead to a further SAR in only 20% of cases and no increase in severity occurred [36]. For this reason, children with mild SAR to Hymenoptera stings do not need to be subject to VIT. 

## Side effects of specific immunotherapy 

In specific immunotherapy with Hymenoptera venom (VIT) local reactions frequently occur, particularly in the initial phase, but unspecific general reactions, like fatigue and lassitude, have also been observed. We would like to focus the aspect of SAR as a side effect of SIT: In prospective multi-center studies analyzing a large number of patients 12% of 1,410 [1^5^] and 20% of 840 [17] patients developed SAR during SIT. The majority of reactions was mild to moderate. When mild cases where no intervention was necessary are not considered, the percentage of affected patients is 6.7% [17] and 8.4% [31], respectively. 

The severity of the reaction to stings before the start of therapy does not influence the SAR during VIT [15, 17, 31]. For the association between gender and SAR as side effects of VIT the data are contradictory: If all SAR were taken into consideration, women were affected more frequently [17]; if the focus was put on severe reactions, no difference between both genders was observed [3^1^]. 

The following risk factors for SAR during VIT have been described:

- The most important risk factor is VIT with honey bee venom, which is significantly worse tolerated than treatment with wasp venom [15, 17, 19, 31].
- During dose increase SAR occur more frequently than during maintenance therapy [17.
- BSTC is correlated with more severe SAR during the dose increase phase, with this effect being remarkably more pronounced in treatment with wasp venom than in therapy with honey bee venom [31].
- Mastocytosis [23].
- IgE antibodies specific for Hymenoptera venom [31].
- Rapid dose increase [17, 31]. In this context it has to be noted that in retrospective single-center studies with high numbers of cases at least VIT with wasp venom was better tolerated when the dose was increased rapidly as compared to a slow dose increase [4]. It has to be taken into consideration, however, that the validity of prospective and multi-centric studies is higher.
- Antihypertensive therapy [31].

When only the role of b-blocker treatment during VIT was examined, no effect in patients with cardiovascular diseases was shown [2^1^]. Concerning discontinuation the above-mentioned is valid. Therapy with ACE inhibitors did not increase the frequency of systemic reactions to VIT either [37]. The risk-increasing effect of antihypertensive therapy could not be attributed to a certain agent and will primarily reflect the increased risk when underlying cardiovascular diseases are present. On no account it should be concluded from these data that these kinds of drugs should simply be discontinued. In fact, cardiovascular diseases should be optimally controlled when VIT is carried out. Only ACE inhibitors should be avoided, because, when VIT does not provide sufficient protection, they increase the risk for severe SAR. 

Particularly in cases of honey bee venom allergy and in the presence of further individual risk factors ultra-rush protocols are not advisable. Although a slower, conventional dose increase (carried out over several weeks) has proven to be better tolerated than rush protocols (carried out over only several days), the broad use of out-patient dose increase regimens cannot be recommended without restrictions. Conventional dose increase can also lead to SAR, and the follow-up as well as the management of such reactions are easier to carry out in an in-patient setting. 

In case of repeated SAR a dose increase can often successfully be achieved using certain procedures [30]. The pre-treatment with antihistamines can reduce side effects that are restricted to the skin, but cannot prevent SAR in general. If promotive factors, like infections, extreme physical or mental stress and mastocytosis cannot be found or eliminated, a change in the dose increase regimen, a transient dose reduction and a subsequent increase of the maintenance dose to ≥ 200 µg (or even higher, if appropriate) can be attempted. The pre-medication with anti-IgE antibodies is effective, but at present can only be carried out as an individual treatment attempt. If this is not successful or possible, therapy is continued with the highest tolerated dose, if this is at least 50 µg. 

## Treatment failure 

Not all patients are protected under VIT. In some studies a diagnostic sting provocation carried out for therapy control lead to a SAR in up to 25% of patients [26]. The evaluation of 1,071 of our patients showed that 7.7% of 1,204 sting provocation tests lead to a SAR [13]. 

The problem is that to date only single-center studies with partly small numbers of cases have been published, and thus, selection effects might influence the results. Another difficulty is that different authors use different definitions of recurring SAR in sting provocations: many authors have interpreted only subjective symptoms as this kind of reaction. 

Based on the available data several risk factors for treatment failure can be identified:

- SIT with honey bee venom is less effective than SIT with wasp venom [9, 13, 19].
- Only in patients with wasp venom allergy increased BSTC has been proven to be a risk factor [9].
- Higher maintenance doses are more effective than lower doses [7, 27].
- SAR as side effects of SIT [13, 18].
- Mastocytosis [23].

The univariate analysis of our own data did not show any influence of age, gender, mast cell tryptase or severity of sting reaction before therapy in the sting provocation results [13]. 

In patients with a particularly increased risk for therapy failure or in cases of therapy failure VIT is carried out with an increased maintenance dose – usually 200 µg is an appropriate dose. Examples for this are honey bee venom allergy in beekeepers as well as presence of a severe cardiovascular disease or mastocytosis. When SAR still occur despite VIT, the maintenance dose is also increased, if necessary even to > 200 µg of the causative insect venom. 

**Table 1:** Increased exposure to Hymenoptera (examples)

Beekeepers and their family members or neighbors
Work in a fruit or bakery shop, as a ground worker, gardener, fire fighter or farmer
Outdoor leisure activities like working in the garden, swimming, golfing or cycling
Motorcycling

**Table 2:** Severity of anaphylactic reactions*: classification according to Ring and Meßmer [23].

Stage I	Generalized reactions limited to the skin (e.g. pruritus, flush, urticaria, angioedema)
Stage II	Mild-to-moderate pulmonary, cardiovascular and/or gastrointestinal symptoms (e.g. nausea, cramps, rhinorrhea, hoarseness, dyspnea, tachycardia with increase in heart rate ≥ 20/min, hypotension with drop in systolic blood pressure ≥ 20 mmHg)
Stage III	Anaphylactic shock (clinical symptoms: bronchospasm, vomiting, spontaneous defecation/urination, mostly loss of consciousness)
Stage IV	Cardiac arrest

*Classification according to the most severe symptom, occurrence of lower stage symptoms is optional.
